# Genetic determinants of severe COVID-19 in young Asian and Middle Eastern patients: a case series

**DOI:** 10.1038/s41598-023-47718-0

**Published:** 2023-11-20

**Authors:** Beshr Abdulaziz Badla, Mohamed Samer Hanifa, Ruchi Jain, Maha El Naofal, Nour Halabi, Sawsan Yaslam, Sathishkumar Ramaswamy, Alan Taylor, Roudha Alfalasi, Shruti Shenbagam, Hamda Khansaheb, Hanan Al Suwaidi, Norbert Nowotny, Rizwana Popatia, Abdulla Al Khayat, Alawi Alsheikh-Ali, Tom Loney, Laila Mohamed AlDabal, Ahmad Abou Tayoun

**Affiliations:** 1 College of Medicine, Mohammed Bin Rashid University of Medicine and Health Sciences, Dubai Health, Dubai, UAE; 2 Al Jalila Genomics Center of Excellence, Al Jalila Children’s Specialty Hospital, Dubai Health, Dubai, UAE; 3 Medical Education and Research Department, Dubai Health, Dubai, UAE; 4 Institute of Virology, University of Veterinary Medicine Vienna, Vienna, Austria; 5 Al Jalila Children’s Specialty Hospital, Dubai Health, Dubai, UAE; 6 Dubai Health, Dubai, UAE; 7 Medical Affairs Department, Rashid Hospital, Dubai Health, Dubai, UAE; 8 Center for Genomic Discovery, Mohammed Bin Rashid University of Medicine and Health Sciences, Dubai Health, Dubai, UAE

**Keywords:** Genomics, Mutation, Sequencing, Molecular medicine, Pathogenesis, Risk factors

## Abstract

Studies of genetic factors associated with severe COVID-19 in young adults have been limited in non-Caucasian populations. Here, we clinically characterize a case series of patients with COVID-19, who were otherwise healthy, young adults (N = 55; mean age 34.1 ± SD 5.0 years) from 16 Asian, Middle Eastern, and North African countries. Using whole exome sequencing, we identify rare, likely deleterious variants affecting 16 immune-related genes in 17 out of 55 patients (31%), including 7 patients (41% of all carriers or 12.7% of all patients) who harbored multiple such variants mainly in interferon and toll-like receptor genes. Protein network analysis as well as transcriptomic analysis of nasopharyngeal swabs from an independent COVID-19 cohort (N = 50; 42% Asians and 22% Arabs) revealed that most of the altered genes, as identified by whole exome sequencing, and the associated molecular pathways were significantly altered in COVID-19 patients. Genetic variants tended to be associated with mortality, intensive care admission, and ventilation support. Our clinical cases series, genomic and transcriptomic findings suggest a possible role for interferon pathway genes in severe COVID-19 and highlight the importance of extending genetic studies to diverse populations to better understand the human genetics of disease.

## Introduction

Since the start of the COVID-19 pandemic, as of the November 8, 2023, there have been over 771,820,937 confirmed cases of COVID-19 including 6,978,175 deaths worldwide^1^. Of these cases and deaths, 1,067,030 cases and 2349 deaths have been reported in the UAE^2^. Several risk factors have been associated with COVID-19 disease severity, including age, sex, smoking, ethnicity, and underlying health conditions like hypertension, diabetes, and cardiovascular disease^3^. Although variable and ranging from asymptomatic to severe, COVID-19 severe clinical presentation is relatively rare in young adults. Therefore, studies have focused on this age group to identify potential genetic determinants of severe COVID-19, although most such studies have been enriched for patients of Caucasian ancestry. Limited studies have focused on patients of other ancestries, such as those of Middle Eastern or Asian origins.

Up to date, the majority of genetic investigations have identified significant genome-wide associations involving loss-of-function mutations in immune response pathway. Zhang et al. reported deleterious mutations in 13 genes involved in type I interferon (IFN) pathway in COVID-19 patients with severe outcomes^4^. Other studies reported associations between *TLR7* and *IFNAR2* loss of function and severe COVID-19^5–7^. Another comprehensive study of 5,085 severe disease cases and 571,737 controls suggested that TLR7-mediated genetic predisposition to severe COVID-19 may be a dominant or co-dominant trait, an observation that cannot be made in cohorts limited to male participants^8^. Patients with severe COVID-19 outcomes were shown to have highly dysregulated expression of chemokine and interferon-related genes^9^. Evidence that SARS-CoV-2 causes Kawasaki-like, multi-system inflammatory disease in children with laboratory-confirmed COVID-19^10–12^ suggests that host variables controlling the immune system and host-virus interactions could contribute to COVID-19 etiology and pathogenesis.

**Figure 1:**
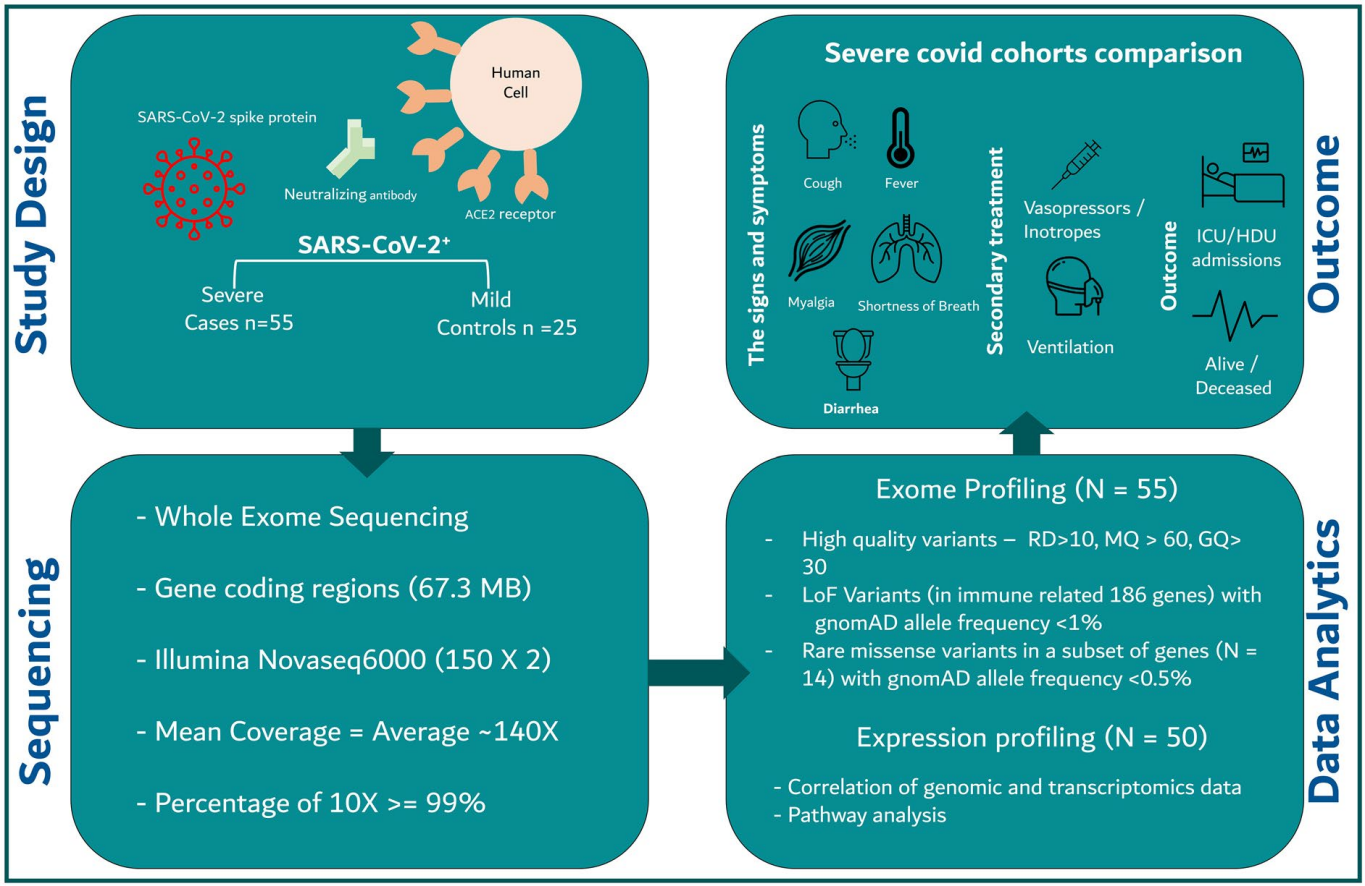
Graphical representation of study design, participants, sequencing protocol, bioinformatic analysis, genomic, transcriptomic and clinical characterization of patients with severe COVID-19. *SARS-CoV-2* Severe acute respiratory syndrome coronavirus 2, *ACE2* Angiotensin converting enzyme 2, *LoF* Loss of function, *gnomAD* Genome Aggregation Database, *ICU* Intensive Care Unit, *HDU* High Dependency Unit.

We aimed to assess these genetic associations with severe COVID-19 outcomes in a case series of young patients from generally understudied populations, in Asia and the Middle East. We determine the burden of rare host genetic variants in an otherwise healthy and young patient population recruited in the United Arab Emirates and assess whether they are associated with clinical outcomes of COVID-19 (Fig. 1).

## Results demographic and clinical characteristics of the cohort

**Figure 2:**
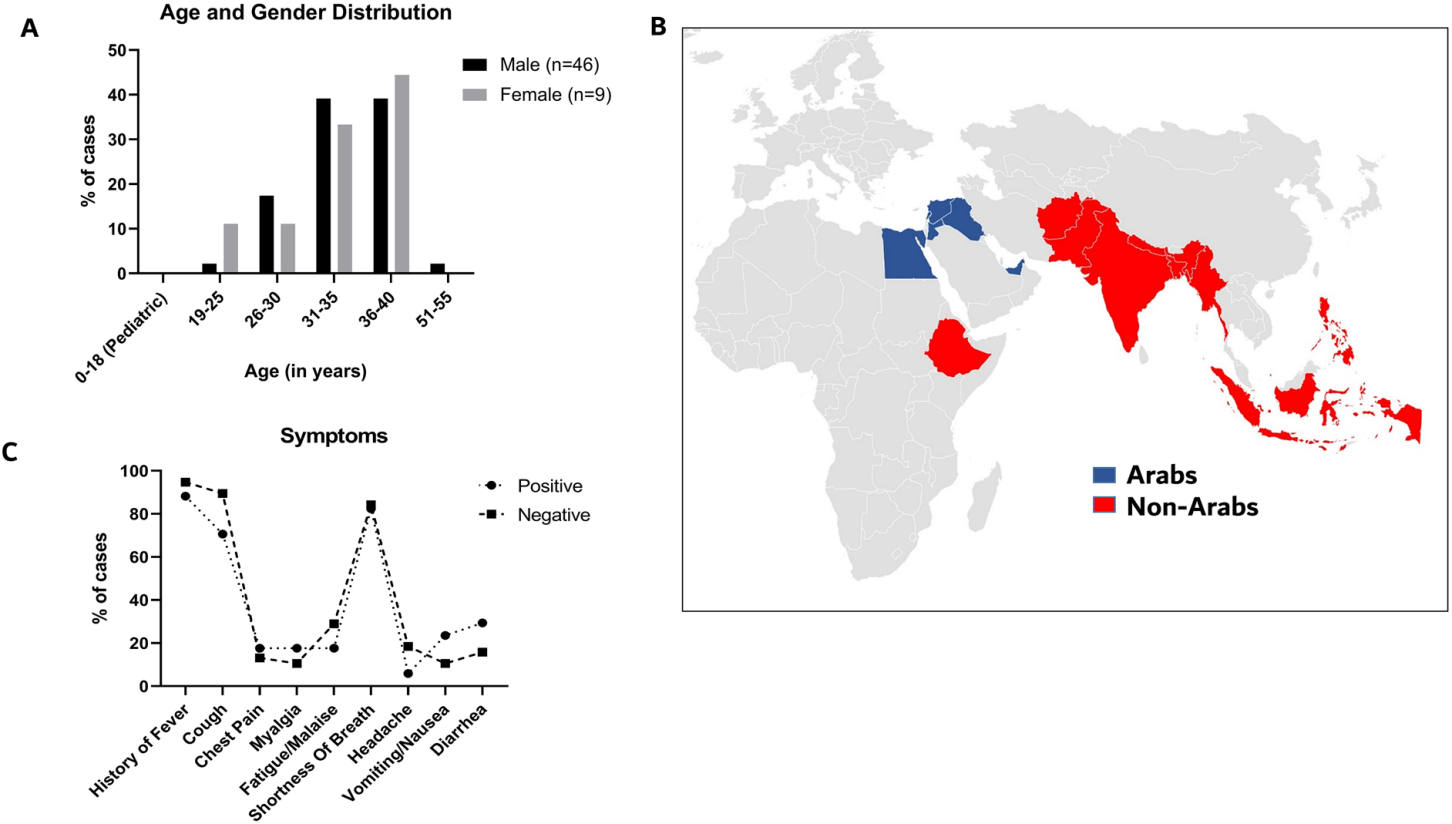
Demographics (age, gender, and country of origin) and clinical symptoms of patients with severe COVID-19. (**A**) Distribution of patients age and gender, X-axis represents age (in years), and Y-axis represents percentage of cases. Grey bars represent females while black bars represent males. (**B**) Geographical map representing patients’ origins or nationalities (**C**) Percentage of patients with major symptoms in each grouping (Positive = patients with genetic findings; Negative = patients without genetic findings). X-axis represents symptoms, and Y-axis represents percentage of cases.

The cohort with severe COVID-19 consisted of 55 patients in total, of whom 83.6% were male (Table 1), with an average age of 34 years (range: 22 to 51 years; SD = 5.0 years) (Table 1 and Fig. 2A). Most patients (81.8%) were Asians, while the remaining were Arabs, with an overall representation from 16 countries in Asia, the Middle East, and the North African region, including the UAE, Jordan, Lebanon, Syria, Palestine, Egypt, Iraq, India, Nepal, Bangladesh, Indonesia, Afghanistan, Myanmar, Pakistan, Philippines, and Ethiopia (Fig. 2B).

**Table 1:** Description of severe COVID-19 cohort (N = 55).

Variable	Mean (SD)
Age	34.13 (5.0)
BMI	29.4 (5.7)
Vitals (on admission)	
Temperature	38.2 °C (1.08)
Heart rate (beats per minute)	108.4 (18.6)
Respiratory RATE (breaths per minute)	24.9 (7.8)
Blood pressure—systolic (mmHg)	125.3 (15.6)
Blood pressure—diastolic (mmHg)	77.82 (13.9)
Oxygen saturation %	90.13 (7.92)
Number of patients with co-morbidities N (%)	
0	46 (83.6%)
1	8 (14.5%)
2	1 (1.8%)

Arabs: UAE, Jordan, Lebanon, Syria, Palestine, Egypt, and Iraq.

Non-Arabs: India, Nepal, Bangladesh, Indonesia, Afghanistan, Myanmar, Pakistan, Philippines, and Ethiopia.

Vitals were measured on admission; on average, temperature was 38.2 °C (SD = 1.1), heart rate was 108 beats per minute (SD = 19), respiratory rate was 25 breaths per minute (SD = 8), systolic blood pressure (BP) was 125 mmHg (SD = 16), and diastolic BP was 78 mmHg (SD = 14), SpO2 levels 90.13 (SD = 7.92). Most patients (83.6%) had no co-morbidities (e.g., diabetes, hypertension, asthma), and 14.5% had only one co-morbidity (Table 1).

We recorded the patient’s symptoms on admission and throughout their stay in the hospitals (Fig. 2C). The most prevalent symptoms within the severe cohort (N = 55) were fever (92.7%), cough (83.6%), and shortness of breath (83.6%). Fatigue/malaise, diarrhea, vomiting, headache, chest pain, and myalgia were observed in 25.5%, 20%, 14.5%, 14.5%, 14.5% and 12.7%, respectively. No patients had any vascular symptoms (like bleeding or lymphadenopathy), skin ulcers, conjunctivitis, or seizures.

### Genetic findings and enrichment analysis

Whole exome sequencing and targeted analysis of 186 immune-related genes (“Methods” section) identified rare putative loss of function variants in 9 patients (16.4%), and rare, likely deleterious missense variants in 12 patients (21.8%). Seven patients (12.7%) harbored multiple rare heterozygous variants, including one patient (COVGEN-3) with two loss of function variants in *TLR4* and *IRAK3*, and another patient (COVGEN-6) with three such truncating variants in *IFI44, TLR6,* and *IFNA4* (Table 2).

**Table 2:** Genetic findings in young patients with severe COVID-19.

Case ID	Chromosome position	Gene(s)	Transcript	cDNA	Protein effect	Zygosity	Effect	Impact
COVGEN-3	chr9:120474928	*TLR4*	NM_138554.5	c.526_544del	p.Asn176Phefs*27	Het	LoF	N/A
	chr12:66605379	*IRAK3*	NM_007199.3	c.588 + 2T > C	p.?	Het	LoF	N/A
COVGEN-6	chr1:79126263	*IFI44*	NM_006417.5	c.1037dupC	p.His347Serfs*4	Het	LoF	N/A
	chr4:38830867	*TLR6*	NM_006068	c.228_229insT	p.Glu77Ter	Het	LoF	N/A
	chr9:21187471	*IFNA4*	NM_021068	c.60T > A	p.Cys20Ter	Het	LoF	N/A
COVGEN-9	chr12:56743250	*STAT2*	NM_005419.4	c.1301C > G	p.Thr434Arg	Het	Rare Missense	SIFT: Deleterious PolyPhen2: Probably damaging
COVGEN-11	chr21:34625115	*IFNAR2*	NM_001289125.3	c.689T > C	p.Leu230Pro	Het	Rare Missense	SIFT: Tolerated PolyPhen2: Probably damaging
COVGEN-14	chr12:64890807	*TBK1*	NM_013254.4	c.1839G > T	p.Leu613Phe	Het	Rare Missense	SIFT: Tolerated PolyPhen2: Probably damaging
COVGEN-18	chr9:21206844	*IFNA10*	NM_002171.2	c.253C > T	p.Gln85Ter	Het	LoF	N/A
	chr21:34621056	*IFNAR2*	NM_001289125.3	c.437A > G	p.Asn146Ser	Het	Rare Missense	SIFT: Tolerated PolyPhen2: Benign
COVGEN-25	chr14:94567118	*IFI27L1*	NM_206949.3	c.61 + 1G > A	p.?	Het	LoF	N/A
	chr19:4816616	*TICAM1*	NM_182919.4	c.1774G > A	p.Gly592Arg	Het	Rare Missense	SIFT: Deleterious PolyPhen2: Benign
COVGEN-28	chr1:235929421	*LYST*	NM_000081.4	c.6079G > C	p.Val2027Leu	Het	Rare Missense	SIFT: Deleterious PolyPhen2: Benign
COVGEN-32	chr12:56742818	*STAT2*	NM_005419.4	c.1466C > T	p.Pro489Leu	Het	Rare Missense	SIFT: Deleterious PolyPhen2: Probably damaging
	chr12:56749951	*STAT2*	NM_005419.4	c.250C > A	p.Gln84Lys	Het	Rare Missense	SIFT: Tolerated PolyPhen2: Benign
COVGEN-79	chr21:34809240	*IFNGR2*	NM_005534.4	c.985G > T	p.Glu329Ter	Het	LoF	N/A
COVGEN-82	chr1:79116171	*IFI44*	NM_006417.5	c.291dupA	p.Glu98Argfs*7	Het	LoF	N/A
	chr1:235929421	*LYST*	NM_000081.4	c.6079G > C	p.Val2027Leu	Het	Rare Missense	SIFT: Deleterious PolyPhen2: Benign
COVGEN-89	chr19:4817579	*TICAM1*	NM_182919.4	c.811C > T	p.Pro271Ser	Het	Rare Missense	SIFT: Tolerated PolyPhen2: Benign
COVGEN-90	chr1:235929421	*LYST*	NM_000081.4	c.6079G > C	p.Val2027Leu	Hom	Rare Missense	SIFT: Deleterious PolyPhen2: Benign
COVGEN-91	chr2:163136505	*IFIH1*	NM_022168.4	c.1641 + 1G > C	p.?	Het	LoF	N/A
COVGEN-94	chr2:163124674	*IFIH1*	NM_022168.4	c.2730C > A	p.Cys910Ter	Het	LoF	N/A
	chr21:34713445	*IFNAR1*	NM_000629.3	c.341G > C	p.Trp114Ser	Het	Rare Missense	SIFT: Tolerated PolyPhen2: Probably damaging
	chr19:4816616	*TICAM1*	NM_182919.4	c.1774G > A	p.Gly592Arg	Het	Rare Missense	SIFT: Deleterious PolyPhen2: Benign
COVGEN-100	chr9:21239446	*IFNA14*	NM_002172.3	c.489delC	p.Trp164Glyfs*9	Het	LoF	N/A
COVGEN-127	chr21:34707884	*IFNAR1*	NM_000629.3	c.131A > C	p.Asn44Thr	Het	Rare Missense	SIFT: Tolerated PolyPhen2: Benign

**Figure 3:**
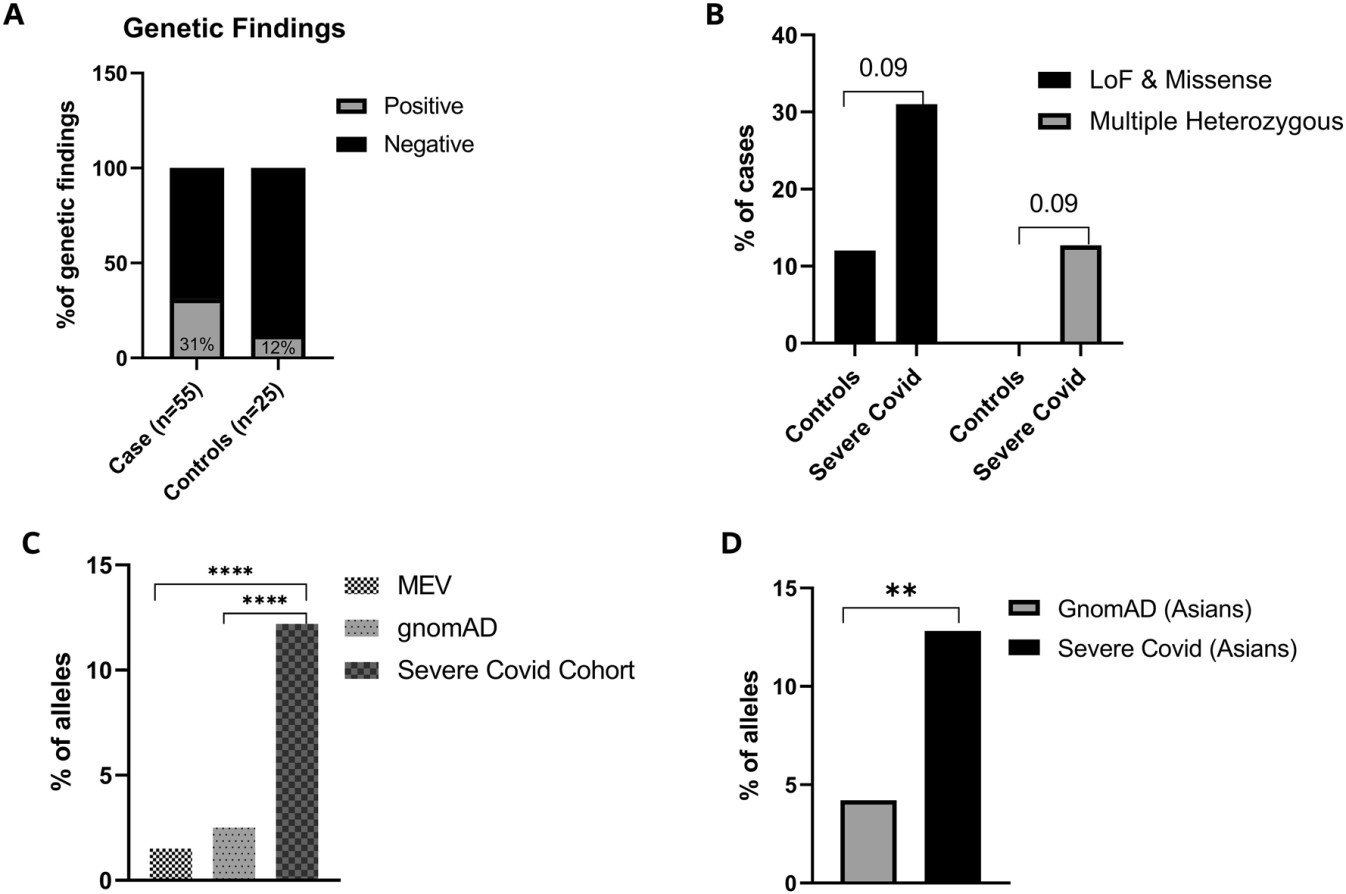
Genetic findings and burden of immune-related loss of function (LoF) and missense variants in patients with severe COVID-19. (**A**) Genetic findings in cases and controls. X-axis represents cases and controls, Y-axis represents % of genetic findings. Grey filled bars represents positive genetic findings and black represents negative genetic findings. (**B**) Percentage of patients or controls with at least one LoF and/or missense variants (black filled bars), or with multiple heterozygous (> 1) variants (Grey filled bars). X-axis represents cases and controls; Y-axis represents percentage of each group. (**C**) Frequency of truncating variants in the 10 genes identified in the severe COVID-19 cohort relative to different populations, y axis representing % of alleles. (**D**) Frequency of truncating variants in Asian patients with severe COVID-19 relative to South and East Asians in gnomAD, y axis representing % of alleles. *P < .05; **P < .01; ***P < .001; ****P < .0001 by the Mann–Whitney test. *gnomAD* The Genome Aggregation Database, *MEV* Middle East Variome Database.

Overall, 17 out of 55 patients with severe COVID-19 (31%; 95% CI, 20.3–44.0%) had at least one rare missense or truncating variant in immune-related genes and 7 (12.7%; 95% CI, 6.7% -31.6%) had multiple such variants. On the other hand, a similar analysis on 25 patients with mild/asymptomatic COVID-19, recruited in the Middle East, identified rare variants in 3 individuals (12%) (Supplementary Table 1), while none of those patients had multiple rare heterozygous variants. This finding suggests that more patients tend to carry rare immunogenetic variants relative to this control group though this finding did not reach statistical significance (*P* = 0.09, Two-sided Fisher’s Exact Test) (Fig. 3A,B). Principal component analysis confirmed the genetic diversity and ancestry composition of the study cohorts while highlighting the close resemblance between the asymptomatic controls and severe COVID-19 patients (Supplementary Fig. 1). We then assessed whether the 10 immune-related genes (*TLR4, IRAK3, IFI44, TLR6, IFNA4, IFNA10, IFI27L1, IFNGR2, IFIH1, IFNA14*) in which we identified 12 truncating variants in 9 patients (Table 2), and which have a similar burden of synonymous variation between cases and controls (data not shown), tolerate truncating variants in the general population databases such as gnomAD and MEV database^14,15^, which is a combined set of exome sequencing data from the Greater Middle East (GME) variome project (N = 1111)^16^ and a Qatari cohort (N = 1005)^17^. If those genes are tolerant to truncating variants in the general population, then such variants will be randomly identified in any group of individuals, including patients with severe COVID-19. However, the same immune-related genes were not significantly enriched for truncating variants in the gnomAD and MEV databases (*P* < 0.0001; Mann–Whitney test), further suggesting a possible enrichment of loss of function variants in those genes in our severe COVID-19 case series (Fig. 3C). Given that patients with severe COVID-19 in our study were primarily Asians (81.8%; Fig. 2A), we performed a similar analysis focusing only on individuals of Asian origin in our severe cohort and gnomAD and found an enrichment of truncating variants in the Asian disease cohort (Fig. 3D). Altogether, while this analysis suggest an association between deleterious rare variants in immune-related genes and severe COVID-19, similar analyses in larger cohorts are needed to corroborate these findings.

### Association of genetic variants with severe COVID-19

**Figure 4:**
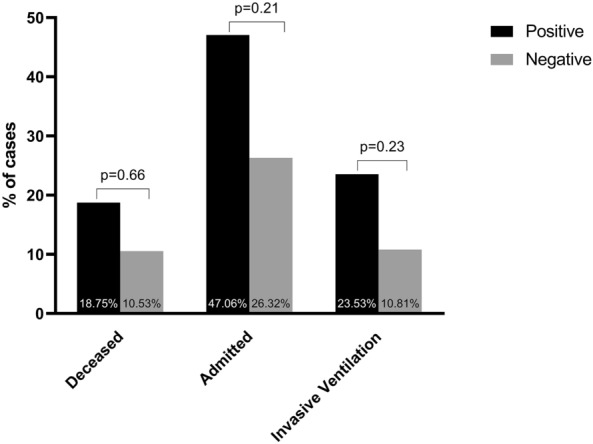
Possible association between rare genetic variants and COVID-19 severity outcomes. Mortality, intensive care unit (ICU)/ high dependency unit (HDU) admission, and ventilation support rates tended to be higher in patients with rare genetic variants (positive) relative to those without such variants (negative). However, none of those associations reached statistical significance.

When combining all the positive genetic findings in the severe cohort into one group (severe-positive cohort) and comparing it to the severe group without such findings (severe-negative cohort), we found that the intensive care unit (ICU)/high dependency unit (HDU) admission rates (47.1% vs. 26.3%), mortality rate (18.8% vs. 10.5%), and invasive ventilation support rate (23.5% vs. 10.8%) were numerically higher (almost doubled) in patients with genetic findings relative to those without (Fig. 4). However, these three relationships did not reach statistical significance, as we were most likely underpowered to find significant associations for rare genetic variants in our small cohort.

Among all 27 rare alleles identified in the patients with severe COVID-19, 20 alleles (74%) were in interferon-pathway-related genes, while 6 alleles (22.2%) were in bacterial infection susceptibility genes. *LYST* had the highest number of alleles (14.8%), followed by *STAT2* and *TICAM1* (11.11% each), and *IFNAR1* and *IFNAR2* (7.4% each) (Supplementary Table 2).

### Pathway and protein network analysis

**Figure 5:**
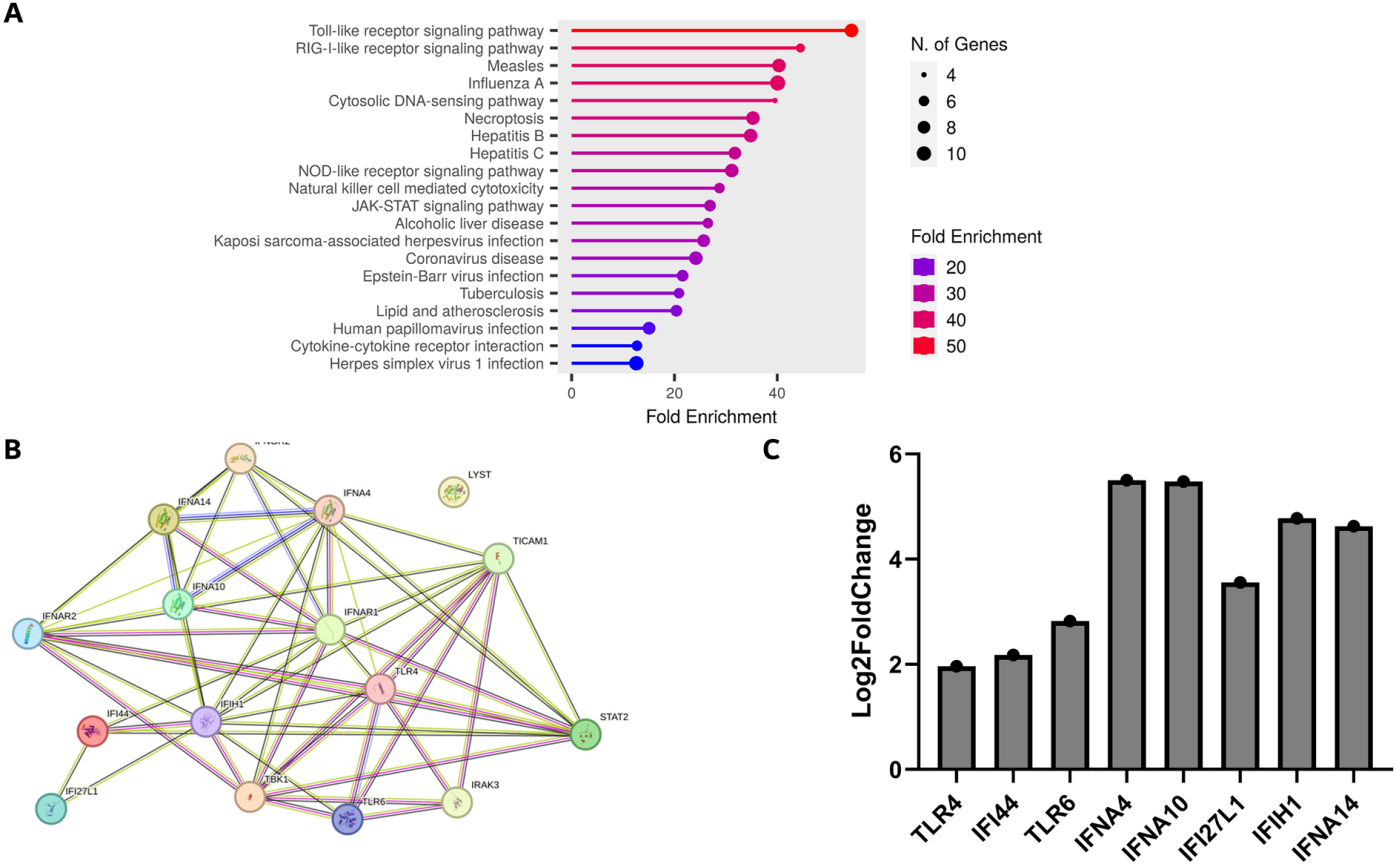
Pathway analysis and transcriptomic profiling. (**A**) Pathway enrichment analysis was performed for the genes with rare variants in severe COVID-19 patients. X-axis represents fold enrichment (the percentage of genes belonging to a pathway, divided by the corresponding percentage in the background); size of the circle represents number of genes enriched in a pathway. (**B**) Protein–Protein Interaction (PPI) network representation of the genes with rare variants in severe COVID-19 patients. Nodes represent proteins, and edges represent confidence of interaction based on functional and curated evidence (See “Methods” section). (**C**) Log2fold change value with adjusted *p value* < 0.001 of genes dysregulated in transcriptomic analysis when compared with controls.

Genes with enriched variants belonged to the Toll-like receptor signaling, RIG-I-like receptor signaling, and NOD-like receptor signaling pathways as the main pathways altered in patients with severe COVID-19 (Fig. 5A). We obtained a protein–protein interaction (PPI) network^18^, based on experimental evidence and expert-curated databases, which showed 16 nodes representing proteins and 56 edges representing confidence level, and a significant PPI enrichment (*P* < 1.0–16) (Fig. 5B). Interactions among *IFNAR2, IFNA4, IFNA14, TBK1, TLR6, TICAM1, TLR4, IFNA10, IFNAR1* indicate the role of IFN-mediated immune responses.

### Overlap of genomic and transcriptomic determinants in patients with COVID-19

To further validate our genetic findings, we assessed the expression of the 16 genes, wherein we identified likely deleterious variants, in an independent cohort of COVID-19 patients (N = 50; 42% Asians and 22% Arabs). Whole transcriptome sequencing data from this independent cohort showed that 8 out of the above 16 genes were significantly dysregulated in COVID-19 patients when compared to controls (N = 32). Specifically, these genes included the interferon and the Toll-like receptor genes: *TLR4, IFI44, TLR6, IFNA4, IFNA10, IFI27L1, IFIH1, IFNA14* (Fig. 5C).

## Discussion

Consistent with the critical role of IFN in viral immunity, our analyses identified several likely deleterious variants in IFN genes and related pathways in a case series of young patients with severe COVID-19 (Table 2). There were variants either in genes coding for IFNs alpha and lambda (*IFNA4* (OMIM 147,564), *IFNA10* (OMIM 147,577), *IFNA14* (OMIM 147,579)), IFN alpha and gamma receptors (*IFNAR1* (OMIM 107,450), *IFNAR2* (OMIM 602,376), *IFNGR2* (OMIM 147,569), *STAT2* (OMIM 600,556), *TBK1* (OMIM 604,834), *TICAM1* (OMIM 607,601), Toll-like receptors (*TLR4* (OMIM 603,030), *TLR6* (OMIM 605,403)) or IFN stimulated genes that code for IFN-induced proteins (*IFI27L1* (OMIM 611,320), *IFI44* (OMIM 610,468), *IFIH1* (OMIM 606951)). Several studies identified variants in Interferon pathway genes in patients with COVID-19. Amado-Rodriguez et al., found that patients with the TT variant (rs199076) in the *IFIH1* had an attenuated inflammatory response to severe SARS-CoV-2 infection^19^. *IFIH1* encodes MDA5, an intracellular sensor of viral RNA that triggers the innate immune response, which was found to be upregulated in cell lines when infected by SARS-CoV-2^20^. Genome wide association studies reported an intronic variant (rs2236757) in the *IFNAR2* gene and found evidence that low expression of *IFNAR2* is associated with severe COVID-19^21^. Other genes in interferon family include *IFI44*, which was also found to be upregulated in SARS-CoV-2 infected primary normal human bronchial epithelial cells^22^, *STAT2* which is critical in activating the transcription of IFN-induced genes in response to IFN stimulation, *TBK1*, a known inducer of innate antiviral type 1 IFNs^23^, and *TICAM1,* which acts as a mediator for dsRNA-TLR3-dependent production of IFN-beta^24^. The high number of IFN pathway-related genetic findings in our severely diseased cohort highlights the importance of IFNs in regulating immunity and mounting an effective immune response against COVID-19.

Bacterial infection secondary to COVID-19 viral pneumonia is associated with a higher risk of death in patients with COVID-19^25^. One of the most frequently mutated genes within our positive genetic findings group (3 patients) was the *LYST* gene (OMIM 606,897) including one patient with a homozygous missense variant (Table 2). This gene regulates lysosomal trafficking and cytoplasmic granule synthesis, fusion, and transport^26^. In Chediak-Higashi syndrome, caused by pathogenic variants in the *LYST* gene, one morbidity of this syndrome is recurrent bacterial infection(s)^26^. Toll‐like receptors (TLRs) are crucial components in the initiation of innate immune responses and triggering the production of pro-inflammatory cytokines as well as type I and II interferons system. We found truncating variants in*TLR4* and *TLR6* genes in two patients. *TLR4* is activated by lipopolysaccharides (LPS), a component of gram-negative bacteria^27^, while *TLR6* is activated by diacylated lipopeptides such as lipoteichoic acid found on the cell wall of gram-positive bacteria. Alterations in genes involved in the destruction of bacteria (*LYST*) and bacterial detection by the immune system (*TLR6* and *TLR4*) could possibly increase susceptibility to bacterial infections secondary to viral pneumonia.

Our protein network and the transcriptomic analyses from an independent cohort of severe COVID-19 all support our genetic findings and confirm an important role for the interferon and Toll-like receptors pathways in mediating the response to SARS-CoV-2 in severe COVID-19 patients. Although 8 out of the 16 genes showed altered expression patterns, we cannot rule out possible changes in expression of the remaining genes given that our transcriptomic analysis was performed on nasopharyngeal specimens where those genes might not be abundantly expressed or modulated.

Previous work by our group found a significant enrichment of genetic variants in predominantly Middle Eastern patients with Multisystem Inflammatory Syndrome in Children (MIS-C), which were associated with earlier onset of disease and resistance to treatment compared to a control group with mild SARS-CoV-2 infection^10^. The same set of immune-related genes were analyzed in the MIS-C study and this present study, identifying likely deleterious variants in 16 and 20 genes in severe COVID-19 and MIS-C, respectively. Interestingly, however, variants were overlapping in 6 genes involved in regulating Toll-like receptor signaling pathway and Interferon signaling pathways (*IRAK3, TLR6, IFNA4, IFNAR2, IFI44, IFIH1*), while the majority of variants were in non-overlapping genes. This finding suggests that, while the genetic determinants of MIS-C and severe COVID-19 in young patients can be slightly different, they both share similarly altered pathways converging mainly on the interferon-signaling pathway, which has recently been shown to be disrupted in another patient population with severe COVID-19^10^.

Our study included patients from genetically underrepresented populations (N = 16 countries) in Asia and the Middle East (Table 1), thus complementing previous genetic studies from other commonly investigated populations. Our findings confirm the genetic determinants of severe COVID-19 in young patients of different ancestries^4^.

While we tried to limit COVID-19 risk factors in our sample, overweight patients were not excluded from our sample. Our cohort with severe COVID-19 had a mean (± SD) BMI of 29.4 ± 5.7 kg/m^2^ which has also been associated with disease severity. The relatively small sample size limited the power of our study, which may explain the lack of statistical significance in some of the correlations between the findings of rare genetic variants and disease outcomes. With a larger sample size and a stricter exclusion criterion, future studies of this nature could be more sensitized and powered to find rare genetic variants associated with disease severity. Povysil G et al. could not observe the enrichment of predicted loss-of-function (pLOF) variants in severe cases relative to population controls or mild COVID-19 cases reported by Zhang et al.^28^. Similarly, exome-wide association analyses of COVID-19 outcomes in 586,157 individuals did not find any association of rare variants and interferon pathway^29^. However, heterogenous study populations, different variant filtering criteria, different definitions of COVID-19 severity, age distribution have resulted in lack of uniform findings and identification of overall impact of genetic markers on severe COVID-19 patients.

Genetic sequencing of young, previously healthy patients with bacterial infections secondary to COVID-19 pneumonia and searching for any genetic variants associated with this outcome could unearth and determine variants leading to increased susceptibility to this complication. Efficacy testing of type 1 IFN as a therapy in patients with COVID-19 or patients with mutations in IFN pathways would determine if type 1 IFN is a viable therapeutic target.

## Methods

### Study design and recruitment

Young patients with severe COVID-19 infection (N = 55) were prospectively recruited between November 2020- November 2021, mainly from Rashid Hospital, Dubai Hospital, Latifa Hospital, and Al Jalila Children’s Specialty Hospital. The control group was recruited in the UAE and Jordan, as previously described^10^. This control group was recruited at the same time and included 25 healthy children (68% males; 96% Arabs) who had a SARS-CoV-2 infection confirmed by RT-PCR but were asymptomatic or experiencing mild symptoms. Individuals in the control group were followed up for 12 weeks to ensure that no signs of MIS-C disease were detected^10^.

This cohort study was approved by the Dubai Scientific Research Ethics Committee-Dubai Health Authority (DSREC-07/2021_05) and the institutional review board of the Specialty Hospital, Jordan (Number 5/1/T/104046). Patients (and their guardians) recruited in Dubai or Jordan provided written informed consent for their deidentified data to be used for research, and this study was performed in accordance with the relevant laws and regulations that govern research in both countries. This study followed the Strengthening the Reporting of Observational Studies in Epidemiology (STROBE) reporting guideline^13^.

The inclusion criteria for the severe cohort were defined as bilateral pneumonia with more than 50% of the lungs involved, dyspnea, and SPO_2_ of less than 94% on room air. All patients had evidence of SARS-CoV-2 infection by RT-PCR. Exclusion criteria were applied to all patients with another diagnosis that can affect their illness's course or interfere with the genetic results, such as congenital heart disease, failure to thrive, or other syndromes.

Sociodemographic information of patients such as age, gender, body mass index (BMI), and nationality was collected. Patient vitals, signs and symptoms, co-morbidities, patient status (discharged or deceased), ventilation support status or if they had been admitted to the ICU or HDU were also recorded as measures of outcomes of severe COVID-19 infection. The principal exposure studied was the presence of genetic variants.

Chi-squared Tests and two-sided Fisher’s Exact Tests were used to assess the association between nominal variables. In the 2 × 2 tables, the Chi-squared Test was used if all cells had a number equal to or greater than 5 and Fisher’s Exact Test for cells with small values (less than 5). SPSS software was used for all the analysis (version 25).

### Demographics map

The map (in Fig. 2) was generated using “Microsoft^®^ Excel^®^”. The input information table contained two columns: one for countries and the other for labelling as either “1” for Non-Arab Countries and “2” for Arab countries. Countries included are Non-Arabs: India, Nepal, Bangladesh, Indonesia, Afghanistan, Myanmar, Pakistan, Philippines, and Ethiopia, and Arabs: United Arab Emirates, Egypt, Jordan, Palestine, Syria, Lebanon, and Iraq. A heat map was created in excel by selecting different colors for the two Non-Arab and Arab country groups (i.e. “1” or “2”).

### Whole exome sequencing

Whole Exome Sequencing (WES) was performed at the genomics of Al Jalila Children’s Specialty Hospital. DNA was extracted from peripheral blood cells using standard DNA extraction protocols (Qiagen, Germany). Following fragmentation by ultra-sonication (Covaris, USA), genomic DNA was processed to generate sequencing-ready libraries of short fragments (300–400 bp) using the SureSelect^XT^ kit (Agilent, USA). RNA baits targeting all coding regions were used to enrich whole-exome regions using the SureSelect Clinical Research Exome V2 kit (Agilent, USA). The enriched libraries underwent next-generation sequencing (2 X 150 bp) using the SP flow cell and the NovaSeq platform (Illumina, USA)^30,31^.

### Alignment, variant calling and filtration

Sequence mapping and variant calling were performed using Sentieon’s germline variant calling pipeline (v2021.12.05) (Sentieon Inc., San Jose, California, USA)^32^. Raw reads were aligned to the human reference genome, GRCh37, which includes decoy sequence to improve the efficiency of read mapping (hs37d5). We mapped all reads from each sample with Sentieon—BWA-MEM algorithm. Following alignment, we sorted the resulting BAM files, removed duplicate reads using Sentieon-util and dedup algorithm, and performed indel realignment and quality recalibration with Sentieon-Realigner and QualCal algorithms with the help of Mills, 1000G gold standard and 1000G phase1 indels. Afterwards, SNPs and indels were called using the Sentieon-Genotyper with the default parameters. High-quality sequencing reads with greater than 10X coverage across all coding regions were retained. Variants with Read Depth > 10, Genotypic Quality > 30, Mapping Quality > 60, and allele frequency < 1% in gnomAD genomes and exomes were retained.

For variant filtration and prioritization, we used signature genes (N = 186) implicated in immune responses (Supplementary Table 3), including cellular response to cytokine, cell mediation of immunity, immune signaling pathway, and interferon signaling pathway from reported literature^4,6,33,34^. We used 3 filters to retain a) LoF (Loss of Function) variants with deleterious effects on RefSeq canonical transcripts in the 186 genes, b) homozygous variants across all protein-coding effects in the same genes, and c) rare missense variants in 14 genes previously associated with severe COVID-19^4,6^, with gnomAD frequency < 0.5%, for downstream analysis. We applied similar criteria for Controls (N = 25) and filtered LoF, homozygous, and rare missense variants. Variants were visually verified with the Integrative Genomics Viewer v.2.15.2 and Alamut Visual Plus v.1.7.2

### Enrichment analysis

We performed enrichment analysis for the genes with LoF or truncating variants by calculating the fractions of such variants in those genes in gnomAD^14^ and in MEV^15^ (Middle East Variome Database, created in-house by assembling sequencing data from Qatar^17^, GME (Greater Middle East))^16^ and comparing those fractions with those in patients with severe COVID-19. We removed low confidence variants and applied an allele frequency cutoff of 1% in gnomAD. Since the majority of the cases were Asians (~ 80%) we did a similar comparison using South and East Asian allele frequencies from gnomAD. The aggregate LoF allele fraction was obtained by summing the total LoF allele fraction in each of the above genes (total LoF allele counts divided by the maximum allele number in the database), and Fisher’s Exact t-test was performed using Graph Pad Prism v9.2.0.

Similarly, the burden of functional variants (missense or LoF: nonsense, frameshift, canonical ± 1 and ± 2 at the 5’ donor and 3’ acceptor splice sites) between severe COVID-19 and controls was performed by comparing the proportion of individuals with at least 1 functional allele in each group, and *p*-values were reported.

### Transcriptomic analysis

Whole transcriptome sequencing of RNA extracted from nasopharyngeal samples was previously performed for an independent group of 50 patients with COVID-19 (64% males; 42% Asians and 22% Arabs)^9^. Briefly, RNA libraries, which were prepared for shotgun transcriptome sequencing using the TruSeq Stranded Total RNA Library kit from Illumina (San Diego, CA, USA), were then sequenced using the NovaSeq SP Reagent kit (2 X 150 cycles) from Illumina (San Diego, CA, USA) to generate a minimum of 15 M reads per sample.

We analyzed this transcriptomic data to assess the expression patterns of the 16 genes, identified by exome sequencing, compared with controls (n = 32)^35^. In brief, DESeq2 package was used to perform batch effects and normalization. Gene expression profiling was calculated for control and COVID-19 patients using Wald test and p-values and log2 fold change was extracted. The resulting genes p-value was adjusted using the Benjamini and Hochberg method. Genes with adj *P value* < 0.05 were called as significant.

### Pathway and protein network analysis

ShinyGO 0.77^36^ was used for pathway enrichment analysis with false discovery rate (FDR) cut off 0.05. The STRING database^18^ was then used for protein–protein interaction (PPI) network analysis for those 16 genes. The network view summarizes predicted associations for group of proteins where nodes correspond to proteins, and the edges represent confidence based on available experimental evidence and expert-curated databases. We applied an interaction score of highest confidence (0.9) to avoid any false-positive interactions among proteins.

### Principal component analysis

Variants from 1000 Genomes Project (1KGP) Phase3 data^37^ was downloaded from 1KGP portal (ftp://ftp.1000genomes.ebi.ac.uk) and Qatar1005 data^17^ was downloaded from Qatar Genome portal (QTRG). The 1000 genome population included African ancestry (ACB, ASW, ESN, GWD, LWK, MSL, YRI), Admixed American ancestry (CLM, MXL, PEL, PUR), East Asian ancestry (CDX, CHB, CHS, JPT, KHV), European ancestry (CEU, FIN, GBR, IBS, TSI), and South Asian ancestry (BEB, GIH, ITU, PJL, STU) as described here: https://ftp.1000genomes.ebi.ac.uk/vol1/ftp/README_populations.md.

To normalize the region, both the data were restricted with SureSelect Clinical Research Exome V2 probe which is used for control and severe covid sequencing; After normalization, Principal Component Analysis (PCA) was performed using Plink^38^ with default parameters. In addition, R v4.3.1 was used to show how the control and severe COVID-19 population fits in among global ancestral groups.

### Supplementary Information

Supplementary Legends.

Supplementary Figure S1.

Supplementary Tables.

Supplementary Table S3.

## Data Availability

The genetic variants generated and/or analyzed during the current study are available in ClinVar database (https://www.ncbi.nlm.nih.gov/clinvar/?gr=1&term=SUB12698283%5bSubmitter+Batch), [Accession Numbers: SCV003798461 to SCV003798483].
