# Supplementary material for: Genetic determinants of severe COVID-19 in young Asian and Middle Eastern patients: a case series
Source: Sci Rep. 2023 Nov 20;13:20294. doi: 10.1038/s41598-023-47718-0 (PMC10661561; doi:10.1038/s41598-023-47718-0)
Supplement: Supplementary file 1 — Supplementary Legends. [file 41598_2023_47718_MOESM1_ESM.docx]

**Supplementary Figure 1**. **A**. Principal component analysis (PCA) of controls and severe COVID-19 with 1000 Genome Project Phase3 and QATAR1005 (N = 1005). **B**. PCA plot shows clustering of control (N = 25) and severe COVID-19 (N = 55) groups.
