# Supplementary material for: Genetic determinants of severe COVID-19 in young Asian and Middle Eastern patients: a case series
Source: Sci Rep. 2023 Nov 20;13:20294. doi: 10.1038/s41598-023-47718-0 (PMC10661561; doi:10.1038/s41598-023-47718-0)
Supplement: Supplementary file 3 — Supplementary Tables. [file 41598_2023_47718_MOESM3_ESM.docx]

**Supplementary Table 1:** Variants detected in Control group

| **Case ID** | **Chromosome coordinates** | **Gene(s)** | **Transcript** | **cDNA** | **Protein Effect** | **Zygosity** | **Effect** |
| --- | --- | --- | --- | --- | --- | --- | --- |
| Control-1 | chr19:39760612 | *IFNL2* | NM_172138.2 | c.562C>T | p.Arg188Ter | Het | Stop gained |
| Control-2 | chr19:39760612 | *IFNL2* | NM_172138.2 | c.562C>T | p.Arg188Ter | Het | Stop gained |
| Control-3 | chr2:163136505 | *IFIH1* | NM_022168.4 | c.1641+1G>C | p.? | Het | Splice donor  variant |

| **Supplementary Table 2:** Genes identified with the variant allele frequencies | | | | |
| --- | --- | --- | --- | --- |
|  | | | Frequency | Percent |
|  | Interferon-Pathway Related Genes | *STAT2* | 3 | 11.11% |
|  |  | *TICAM1* | 3 | 11.11% |
|  |  | *IFNAR1* | 2 | 7.41% |
|  |  | *IFNAR2* | 2 | 7.41% |
|  |  | *IFIH1* | 2 | 7.41% |
|  |  | *IFI44* | 2 | 7.41% |
|  |  | *IFI27L1* | 1 | 3.7% |
|  |  | *IFNA4* | 1 | 3.7% |
|  |  | *IFNA10* | 1 | 3.7% |
|  |  | *IFNA14* | 1 | 3.7% |
|  |  | *IFNGR2* | 1 | 3.7% |
|  |  | *TBK1* | 1 | 3.7% |
|  | Potential Bacterial Infection Susceptibility Genes | *LYST* | 4 | 14.8% |
|  | Overlapping Genes between Interferon-Pathway Related Genes and Potential Bacterial Infection Susceptibility Genes | *TLR4* | 1 | 3.7% |
|  |  | *TLR6* | 1 | 3.7% |
|  | Other Genes | *IRAK3* | 1 | 3.7% |
|  | Total* |  | 27 | 100% |
| *Total value represents the number of alleles mutated in total. | | | | |
